# Age-Related Hypercholesterolemia and HMG-CoA Reductase Dysregulation: Sex Does Matter (A Gender Perspective)

**DOI:** 10.1155/2010/420139

**Published:** 2010-05-04

**Authors:** Laura Trapani, Valentina Pallottini

**Affiliations:** Department of Biology, University of Roma Tre, Viale Marconi, 446, 00146 Rome, Italy

## Abstract

Although cardiovascular diseases are less prevalent in premenopausal women than in men, their occurrence in women increases at the onset of menopause, and the loss of female sex hormones contributes to the striking increase in cardiovascular morbidity and mortality in postmenopausal women. We present here a description of age-related disruption of lipid homeostasis, which particularly affects 3-hydroxy 3-methylglutaryl Coenzyme A reductase, the key rate-limiting enzyme in the cholesterol biosynthetic pathway. We further discuss the age- and gender-related dysregulation of this enzyme, providing new evidence for the different mechanisms driving dyslipidemia in elderly men and women. In addition, we introduce pharmacological methods of regulating HMGR and maintaining cholesterol homeostasis.

## 1. Introduction

Aging has been defined as the series of the deteriorative changes occurring during the adult period of life that underlie increased vulnerability to challenges and decreased survival [1]. This deterioration is responsible for both the commonly recognized sequential changes that accompany advancing age and the progressive increase in the chance of disease and death and is usually manifested as a progressive decrease in physiological functions.

Aging is characterized by the loss of homeostasis [2] that leads to changes in the biochemical composition of tissues [3–5], reduced ability to respond adaptively to environmental stimuli [6], and increased susceptibility and vulnerability to diseases [7] including coronary artery diseases (CAD). The term CAD refers to pathologic changes within the coronary artery walls that result in diminished blood flow through these vessels. CAD can cause myocardial ischemia and possibly lead to acute myocardial infarction through three mechanisms—profound vascular spasm of the coronary arteries, formation of atherosclerotic plaques, and thromboembolism. 

Although it is widely accepted that abnormal levels of lipids and/or lipoproteins in blood are modifiable risk factors for CAD [8, 9], the importance of lipid levels as prognostic factors in older adults is controversial. Several studies have suggested that the association between cholesterol concentration and atherosclerotic CAD weakens with age, and that screening and treating older adults for dyslipidemia provides little potential benefit [10, 11]. In contrast, other reports suggest that lipoprotein levels remain a significant risk factor for CAD in the elderly and that treatment of dyslipidemia in the elderly may have a greater impact on CAD mortality than in younger people because the total attributable risk from dyslipidemia is greater in the older age group [12, 13]. 

The mechanisms behind this age-related dyslipidemia are incompletely characterized. Some evidence demonstrates that the causes of age-related disruption of lipid homeostasis include the gradual decline in fractional clearance of LDL with increasing age, the progressively reduced ability to remove cholesterol through conversion to bile acids, and the decreased activity of the rate-limiting enzyme in bile acid biosynthesis, cholesterol 7*α*-hydroxylase (C7*α*OH). Moreover, an interesting hypothesis states that critical changes in cholesterol and lipoprotein metabolism depend on the progressive decrease in growth hormone (GH) secretion, a characteristic feature of aging. GH plays an important role in cholesterol homeostasis by either modulating the expression of hepatic LDLr [14] or controlling the activity of cholesterol 7*α*-hydroxylase [15]. 

CAD is less prevalent in premenopausal women than in men, but its occurrence in women increases at the onset of menopause, and the loss of female sex hormones contributes to the striking increase in the incidence of cardiovascular morbidity and mortality in postmenopausal women. Estrogen replacement therapy results in improved lipoprotein profiles in postmenopausal women, but these improved profiles account for less than half of the cardioprotective effects of estrogen replacement therapy. The cardioprotective effects of estrogens include beneficial changes in plasma lipid levels (increased high-density lipoprotein (HDL), decreased total cholesterol and low-density lipoprotein (LDL), and decreased LDL oxidation) [16, 17], antiplatelet and antioxidant effects, and preservation of endothelium-mediated vasodilation [18, 19]. Providing further evidence for the cardioprotective role of estrogen, studies performed in estrogen-deficient animal models have demonstrated a disruption of lipid homeostasis [20]. 

Cholesterol plays an essential role in the synthesis of new membranes, the turnover of lipids in existing membranes, and the biosynthesis of products such as steroid hormones and bile acids [21]. Cholesterol homeostasis is maintained by a feedback regulatory system that senses the level of cholesterol in cell membranes and modulates both the transcription of genes encoding proteins involved in cholesterol biosynthesis and posttranscriptional events along with the uptake of cholesterol from plasma lipoproteins [22]. Maintenance of cholesterol homeostasis is regulated by both the receptor-mediated endocytosis of LDL by LDL receptors (LDLr) and *de novo* cholesterol synthesis via the rate-limiting enzyme 3-hydroxy-3-methylglutaryl coenzyme A reductase (HMGR) [23]. Because of the pivotal role of HMGR in cholesterol and nonsterol isoprenoid compound biosynthesis, most of the mechanisms controlling cholesterol homeostasis are related to short- and long-term regulation of HMGR. 

To provide new evidence for the different mechanisms driving dyslipidemia in elderly men and women, this review will focus on age-related disruption of lipid homeostasis, and in particular on the age- and gender-related dysregulation of HMGR, the key rate-limiting enzyme in the cholesterol biosynthetic pathway.

## 2. HMGR Regulation in Adults

**Figure 1:**
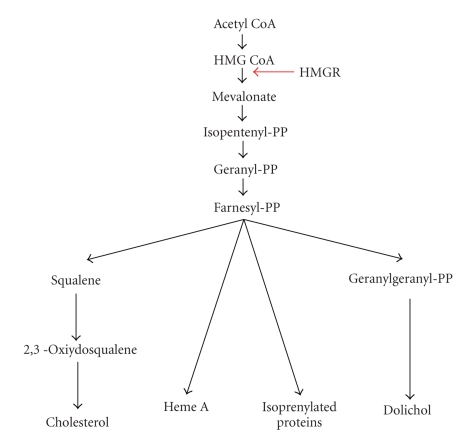
Schematic illustration of the biosynthetic pathway of HMGR end-products.

Cholesterol biosynthesis occurs through a tightly regulated pathway that employs multiple feedback mechanisms to maintain homeostasis [24]. Over the past several decades, much work has focused on the regulation of HMGR, which catalyzes the conversion of HMG-CoA to mevalonate (MVA) through a four-electron oxidoreduction. This reaction is the rate-limiting step in the synthesis of cholesterol and other isoprenoids such as dolichol, isopentenyladenine, which is present in some tRNAs, heme A, ubiquinone, and prenylated proteins such as Ras and Rab proteins (Figure 1) [24]. 

Encoded by the *HMGR *gene located on chromosome 5 of human genome, HMGR consists of a single 888 amino acid polypeptide chain. The N-terminal membrane domain contains 339 hydrophobic residues that span the endoplasmic reticulum (ER) membrane and contains the sterol-sensing domain (SSD), which is responsible for the binding of sterols and other MVA derivatives that accelerate enzyme degradation [25], while the catalytic site is located in the hydro-soluble C-terminal cytoplasmic domain. A linker region (residues 340–459) connects these two portions of the protein [2]. 

Short-term regulation of HMGR is achieved through its phosphorylation and dephosphorylation, both of which can affect enzyme activity. Phosphorylation of residue S872 of HMGR decreases its catalytic activity, and removal of this phosphate results in reactivation [26, 27]. AMP-activated kinase (AMPK) appears to be the major HMGR kinase in the liver, where cholesterologenesis takes place. AMPK is a heterotrimeric serine/threonine kinase consisting of a catalytic *α* subunit and regulatory *β* and *γ* subunits [28]. AMPK is activated by phosphorylation of the *α* subunit at a specific threonine residue (Thr172) [29]. HMGR activation is mediated by its dephosphorylation by protein phosphatase 2A (PP2A), which regulates a significant network of cellular events [30]. 

In addition to this short-term regulation, HMGR is subject to transcriptional, translational, and posttranslational control [31]. These levels of control, which are mediated by changes in intracellular sterol levels and cholesterol uptake by LDLr, can result in changes of over 200-fold in HMGR levels [32]. Both LDLr and HMGR are produced in response to activation of the Sterol Regulatory Element Binding Proteins (SREBPs), and particularly SREBP-2, in the liver [33, 34]. 

Long-term regulation of HMGR is mediated by a pair of membrane-bound proteins, SREBP cleavage activating protein (Scap) and Insulin-induced gene (Insig), which directly bind sterols and thereby sense sterol concentration in the membranes of the ER. As a result of these binding events, both Scap and Insig undergo conformational changes that initiate a series of molecular events blocking Scap's ability to transport SREBPs to the Golgi, terminating cholesterol synthesis and uptake [32]. Furthermore, the intracellular accumulation of sterols induces HMGR to bind Insig, promoting ubiquitination and proteasomal degradation of HMGR [35]. 

Several hormones, including insulin, glucagon, glucocorticoids, thyroid hormone, and estrogen, regulate the expression of hepatic HMGR in animals. Insulin likely stimulates HMGR expression by increasing its rate of transcription, while glucagon opposes this effect. Hepatic HMGR activity undergoes significant diurnal variations due to changes in the levels of immunoreactive proteins, which are primarily mediated by changes in insulin and glucagon levels. Thyroid hormone increases hepatic HMGR levels by acting to increase both transcription and mRNA stability, while glucocorticoids decrease hepatic HMGR expression by destabilizing HMGR mRNA [36]. The effects of estrogen on HMGR expression are still debated. Some studies suggest that estrogens act to increase hepatic HMGR activity primarily by stabilizing HMGR mRNA and that deficiencies in those hormones that act to increase hepatic *HMGR *gene expression lead to elevated serum cholesterol levels [36]. On the other hand, studies using the DLD1 cell line suggest that estrogens induce an early increase in LDLr at both the mRNA and the protein level and later cause decreases in HMGR activity and protein expression [37]. 

Although the mechanisms that regulate cholesterol homeostasis are well known, [22] the literature describing putative physiological sex differences in cholesterol homeostasis-related proteins is limited [38–42]. Furthermore, most of these papers are fragmented and very old, and none of them focus on the mechanisms underlying these sex-related differences. De Marinis and coworkers [43] provided evidence of sex-related physiological differences in proteins involved in cholesterol homeostasis. In particular, activity and expression levels of HMGR are lower in 3-month-old female rats and in 17-*β*-estradiol-treated 3-month-old male rats than in 3-month-old untreated male rats. Moreover, 3-month-old female rats express lower levels of SREBP-2 and higher levels of Insig than their male counterparts. Sex-related variations in expression of these regulatory proteins are functionally consistent with the well-accepted classical model of HMGR behavior [22, 32], and no sex-related differences have been observed in either LDLr expression or cholesterol levels, excluding the involvement of end-product feedback in presence of physiological content of estradiol. The difference in the expression pattern of regulatory proteins in males and females seems to be related to the presence of estrogen, and altered expression of these regulatory proteins drives the sex-related differences in HMGR expression. 

### 2.1. Sex-Related Differences in HMGR Dysregulation during Aging

Due to the serious health-related consequences of aging, significant efforts have been made to provide a more complete understanding of this particular stage of life. Current research aiming to delineate the biological mechanisms of aging has yielded valuable information about the molecular basis of age-related physiological deterioration. One of the critical problems associated with aging is the increased incidence of CAD and, more generally, cardiovascular diseases (CVD). Many risk factors predispose elderly people to develop pathologies related to failure of the heart vasculature, including hypercholesterolemia. Thus, understanding the mechanisms driving increased cellular and plasma cholesterol content during aging is essential in defining specific intervention points. 

During aging, hepatic lipid modifications occur. In particular, studies of 24-month-old male rats showed increased plasma cholesterol levels and increased hepatic cholesterol synthesis accompanied by full activation of HMGR [44, 45], which was dependent on the well-known age-related increase in reactive oxygen species (ROS) [46, 47]. The age-related increase in activation of HMGR has been associated with an increase in ROS [48, 49]. The current model proposes that increased ROS levels result in activation of both p38 and AMPK*α*. In turn, p38 activation may result in an increase in association of PP2A with HMGR, leading to dephosphorylation and increased activation of HMGR. AMPK*α* kinase activity is impaired by the enhanced association of PP2A with HMGR [50]. Moreover, findings in H_2_O_2_-stimulated HepG2 cells demonstrate that the effect of ROS on HMGR dephosphorylation is mediated by activation of the p38/MAPK pathway [45]. 

In addition to the short-term regulation mediated by phosphorylation and dephosphorylation, long-term regulation of HMGR also appears to be affected by aging. Age-related variations in hormone levels and hormone sensitivity induce a decreased ability to maintain homeostatic potential, and these hormonal changes are always associated with changes in the expression or functionality of some molecules. In particular, it has been clearly demonstrated that the age-related decrease in insulin sensitivity induces changes in some factors involved in cholesterol metabolism, such as Insig-1 protein. This age-related reduction in Insig expression results in slower degradation of HMGR [51, 52]. 

While many studies have established that susceptibility to CAD increases with age, little is known about the mechanisms underlying the increased incidence of CAD in postmenopausal women as compared to men of the same age. 

Previous studies have shown that 12-month-old estropausal rats, in which estrogen levels are decreased, have higher levels of plasma cholesterol, increased activation of HMGR, and decreased LDLr membrane exposure than 3-month-old female rats. These changes result in decreased cholesterol uptake and increased cholesterol synthesis, supporting the correlation between hypercholesterolemia, aging, and estropause. Increased activation of HMGR does not depend on an increase in ROS as seen in aged-matched male rats [53]. Instead, HMGR activation seems to be due to decreased activation of AMPK during the period of 17-*β* estradiol deficiency that occurs at the beginning of estropause; this decrease in AMPK activation results in decreased phosphorylation of HMGR. 

Treatment of older female rats with 17-*β* estradiol results in restoration of normal cholesterol levels, decreased activation of HMGR, and increased LDLr exposure on the plasma membrane. Furthermore, while 17-*β* estradiol treatment does not fully restore AMPK activation, AMPK is sufficiently activated in older 17-*β* estradiol-treated female rats to phosphorylate HMGR, reestablishing HMGR activity [54]. This estradiol-induced enhancement in AMPK activation is supported by studies by Schulz and coworkers, who demonstrated that estradiol-mediated AMPK activation was independent of estrogen receptor ligand engagement and involved catechol metabolism of estradiol [55]. 

The decrease in estradiol levels that occurs at the onset of estropause does not affect long-term regulation of HMGR, but mediates short-term HMGR regulation by controlling activation of AMPK. Thus, a relationship exists between changes in estrogen levels and HMGR-related modulation of cholesterolemia in older female rats. The protective role played by estrogens in modulating the lipid profile is mediated not only through increases in plasma HDL, decreases in plasma LDL, and decreased oxidation, but also through regulation of AMPK activation, which inhibits HMGR and cholesterol synthesis.

## 3. Conclusion and Future Perspectives

In elderly men and women, HMGR is highly activated; however, the mechanisms driving dysregulation of HMGR appear to be gender-dependent. Studies of aged male rats suggest that in males, HMGR dysregulation is due to increased association between PP2A and HMGR, which results in increased activation of HMGR. On the other hand, studies of estropausal female rats, in which estrogen levels are decreased, suggest that the menopause-related increase in HMGR activity is caused by the decreased activation of AMPK observed during estrogen deficiency. 

The regulation of HMGR activity has been an attractive target for pharmacological treatment of hypercholesterolemia, the main risk factor for CAD. Consequently, better understanding of the molecular mechanisms that drive dysregulation of HMGR activity and hypercholesterolemia in aged men and women could provide gender-specific targets for treatments to lower plasma cholesterol content, resulting in both prevention and reduction of one of the main risk factors for cardiovascular diseases. 

Decreased cellular cholesterol synthesis leads to a homeostatic response involving up-regulation of cell-surface receptors that bind atherogenic lipoproteins such as LDL and VLDL. These lipoproteins are taken up by the cell and degraded [56], resulting in a reduction in circulating atherogenic lipoproteins. This process helps to explain the clinical usefulness of HMGR inhibitors (statins). 

HMGR inhibition results in not only reductions in cellular and plasma cholesterol levels, but also reductions in other products synthesized through the cholesterol biosynthetic pathway, such as ubiquinone, prenylated proteins, and dolichol. The restoration of HMGR to its normal activation state regulates the physiological synthesis of cholesterol within cells, and restored cellular cholesterol levels are in turn reflected in proper membrane LDLr presence [22]. 

Statins are effective means of primary and secondary prevention of ischemic heart disease (IHD) in middle-aged men; however, proof of the efficacy of statins in preventing development and progression of IHD in women and elderly people is less convincing. In the PROSPER (Prospective Study of Pravastatin in the Elderly at Risk of vascular disease) trial, pravastatin not only had no effect in men and women aged 70–82 years, but also significantly increased the rate of breast cancer in these patients. In the ALLHAT-LLT (Antihypertensive and Lipid-Lowering Treatment to Prevent Heart Attack Trial) trial, pravastatin lowered neither the total number of nonfatal myocardial infarctions and IHD deaths nor total mortality in patients aged 65 years and older and in women [57]. 

Considering the undesirable side effects of statins and the effects of these drugs on other important compounds in addition to cholesterol, development of new pharmacologically active compounds capable of regulating plasma cholesterol content is critical to effectively control this important CVD risk factor. 

Many recent studies have described new compounds able to decrease plasma cholesterol content. Although the exact mechanisms by which these compounds act are unknown, some of these compounds affect expression levels and activity of HMGR. These novel compounds could thus represent the future of hypercholesterolemia therapy and should be studied further. 

Hypercholesterolemia can also be approached using other therapeutic targets; for example, proprotein convertase subtilisin/kexin type 9 (PCSK9) has been implicated as an important regulator of LDL metabolism. PCSK9 belongs to the subtilisin family of serine proteases and is highly expressed in the liver [58]. Secreted PCSK9 modulates LDL levels through posttranslational down-regulation of hepatic LDLr protein [34]. Down-regulation of PCSK9 could thus be effective in decreasing plasma cholesterol content by increasing LDLr levels without affecting activity of HMGR and its end-products. 

In addition, squalene synthase, an enzyme that is downstream of HMGR in the cholesterol synthesis pathway, modulates the first committed step of hepatic cholesterol biosynthesis at the final branching point of the cholesterol biosynthetic pathway. Pharmacologic inhibitors of squalene epoxidase and oxidosqualene cyclase, two enzymes that act downstream of squalene synthase, may thus be useful in reducing plasma LDL content [59]. 

Moreover, the identification of Scap and Insig as sterol-binding proteins in mammalian cells has added a new level of molecular detail to the understanding of regulation of the SREBP pathway and subsequent regulation of HMGR levels, providing new potential targets for pharmacological intervention. 

Much work remains to define the relationship between hormonal changes and their effects on transcription factors and cholesterol metabolism in different physiological and pathological conditions. For example, some studies suggest that estrogens are able to regulate cholesterol homeostasis without directly affecting HMGR [43]. Additionally, more detailed studies are required to define the specific roles of Insig proteins and to determine the metabolic consequences of their reciprocal regulation. In fact, since they are required for feedback regulation of SREBP processing and HMGR degradation, Insigs may represent a new target for pharmacological intervention to maintain blood cholesterol levels within the optimal range. None of the papers we have cited focus on the sex-related differences. 

Thus, although HMGR plays a pivotal role in regulating cholesterol metabolism, future studies should address sex-related differences in the cholesterol biosynthetic pathway to identify new targets for customized pharmacological treatment of hypercholesterolemia.
